# Comparison of Feeding Practices and Growth of Urbanized African Infants Aged 6–12 Months Old by Maternal HIV Status in Gauteng Province, South Africa

**DOI:** 10.3390/nu15061500

**Published:** 2023-03-21

**Authors:** Phumudzo Tshiambara, Marinel Hoffman, Heather Legodi, Tanita Botha, Helen Mulol, Pedro Pisa, Ute Feucht

**Affiliations:** 1 Department of Human Nutrition, Faculty of Health Sciences, University of Pretoria, Prinshof Campus, Pretoria 0084, South Africa; 2 Department of Consumer and Food Sciences, Faculty of Natural and Agricultural Sciences, University of Pretoria, Hatfield Campus, Pretoria 0028, South Africa; 3 Research Centre for Maternal, Fetal, Newborn and Child Health Care Strategies, University of Pretoria, Kalafong Provincial Tertiary Hospital, Pretoria 0001, South Africa; 4 Research Unit for Maternal and Infant Health Care Strategies, South African Medical Research Council, Pretoria 0001, South Africa; 5 Department of Statistics, Faculty of Natural and Agricultural Sciences, University of Pretoria, Hatfield Campus, Pretoria 0028, South Africa; 6 Department of Paediatrics, Faculty of Health Sciences, University of Pretoria, Prinshof Campus, Pretoria 0084, South Africa

**Keywords:** HIV exposure, infants, anthropometry, growth, feeding practices, breastfeeding, nutrition

## Abstract

Appropriate feeding practices are protective against malnutrition and poor growth. We compared feeding practices and growth in HIV-exposed-uninfected (HEU) and HIV-unexposed-uninfected (HUU) between 6-12 months of age in urbanized African infants in South Africa. A repeated cross-sectional analysis was used to determine differences in infant feeding practices and anthropometric measures by HIV exposure status at 6, 9, and 12 months in the Siyakhula study. The study included 181 infants (86 HEU; 95 HUU). Breastfeeding rates were lower in HEU vs. HUU infants at 9 (35.6% vs. 57.3%; *p* = 0.013) and 12 months (24.7% vs. 48.0%; *p* = 0.005). Introduction to early complementary foods was common (HEU = 16.2 ± 11.0 vs. HUU = 12.8 ± 9.3 weeks; *p* = 0.118). Lower weight-for-age Z-scores (WAZ) and head circumference-for-age Z-scores (HCZ) were found in HEU infants at birth. At 6 months, WAZ, length-for-age Z-scores (LAZ), HCZ, and mid-upper-arm circumference-for-age Z-scores (MUACAZ) were lower in HEU vs. HUU infants. At 9 months, lower WAZ, LAZ, and MUACAZ were found in HEU vs. HUU infants. At 12 months, lower WAZ, MUACAZ, and weight-for-length Z-scores (−0.2 ± 1.2 vs. 0.2 ± 1.2; *p* = 0.020) were observed. HEU infants had lower rates of breastfeeding and poorer growth compared to HUU infants. Maternal HIV exposure affects the feeding practices and growth of infants.

## 1. Introduction

Globally 38.4 million people were living with HIV, and 28.7 million were accessing antiretroviral therapy (ART) in the year 2022 [1]. In the high-prevalence country of South Africa, 8.2 million people were living with HIV, including 23.9% of all women of reproductive age in 2021 [2]. The country introduced public health HIV programs, including the prevention of mother-to-child transmission (PMTCT) program in 2002 and the ART program in 2004, to reduce mortality and prevent viral transmission [3,4]. The success of these programs has led to large numbers of infants being born with in utero exposure to maternal HIV infection while remaining HIV-exposed-uninfected (HEU) [5,6]. In Sub-Saharan Africa (SSA) alone, there are more than one million annual births of HEU children [7].

Sub-optimal infant feeding practices negatively impact childhood growth. Therefore the Infant and Young Child Feeding (IYCF) policy emphasizes breastfeeding as a cornerstone for health and survival, including in the context of HIV [8]. This is also in line with the World Health Organization (WHO) recommendations for breastfeeding for at least the first 24 months of life [9], preventing malnutrition, including stunting, underweight, and wasting [10,11,12]. Many women living with HIV still fear that breastfeeding may lead to vertical HIV transmission [13,14], even though this risk is significantly lowered within the context of ART provision [3,15].

To ensure optimal growth, safe and nutritious food should be introduced to infants after the age of 6 months, in addition to continued breastfeeding [16]. However, early introduction of complementary feeding is a very common practice in South Africa (45–87.5%) [17,18,19,20,21,22], which is an important public health concern [23], with stunting (30.5%), wasting (30.3%) and underweight (33.2%) reported already in 4–5-month-old infants [24].

Infants who are HEU are known to be at greater risk of adverse birth outcomes, morbidity, and infections, affecting their growth and development [25,26]. These sub-optimal growth outcomes include being underweight, stunting, or even wasting [10,27]. In addition, HEU infants are also smaller at birth in terms of the mean weight and length [28,29], which may contribute to their poor growth observed as early as three months of age, as documented in multiple African countries (Zambia: lower mean weight 2.9 kg vs. 3.0 kg between 1–16 weeks, lower length in HEU infants at 6 months (64.3 cm ± 1.1) [30]; Zimbabwe: the likelihood of stunting (25%), underweight (55%), and wasting (58%) high in HEU infants [31]; Ethiopia: risk of stunting of 51.9 per 100 person-years infants HIV exposed from conception [32]; and South Africa:10% HEU stunted [33]). Limited research is available on HEU infants that focuses on the complementary feeding introduction phase, especially in terms of having an appropriate HIV-unexposed-uninfected (HUU) comparison group [11,28,34,35,36,37]. Therefore, this study aimed to compare the feeding practices and growth of HEU and HUU infants between 6–12 months of age in Tshwane District, Gauteng Province, South Africa.

## 2. Materials and Methods

### 2.1. Study Design and Setting

This study is a sub-study of the longitudinal Siyakhula cohort study [37], which aims to better understand how the in-utero and early postnatal environments, altered by maternal HIV infection and the treatment thereof, influence infants’ growth trajectories and cognitive development and alter their immune development and function, irrespective of the infants’ own HIV status. For this study, mother-infant dyads who attended the 6 (*n* = 181), 9 (*n* = 166), and 12 (*n* = 155) month follow-up visits were included, with declining numbers due to loss to follow-up and relocation.

### 2.2. Data Collection

All study-related information was collected at the central study site at Kalafong Provincial Tertiary Hospital, Gauteng Province, South Africa [37]. After obtaining informed consent, questionnaires were administered in the participants’ preferred local languages by trained fieldworkers. Socio-demographic information was collected using a structured questionnaire and included maternal age, marital status, level of education, employment status, as well as the infants’ age, sex, and HIV exposure status (with all women living with HIV self-reporting use of ART during and after pregnancy, with the first-line regimen at the time of study being a once-daily fixed-dose combination of tenofovir, emtricitabine, and efavirenz).

Infant growth was assessed by documenting weight (calibrated digital scale; Seca 354, Seca, Hamburg, Germany), length (mechanical infantometer; Seca 416, Seca, Hamburg, Germany), head circumference, and mid-upper-arm-circumference (MUAC) (non-stretchable tape measure; KDS measure, model F10-02DM 2m, Kyoto, Japan), wearing minimal clothing. These measurements were available for the time of birth (except MUAC) for baseline purposes and then at 6, 9 and 12 months as part of study-related procedures. Z-score indices, including weight-for-age (WAZ), length-for-age (LAZ), weight-for-length (WLZ), HC-for-age (HCZ) & MUAC-for-age (MUACZ), were computed using the Intergrowth-21st and WHO Anthro child growth standards v3.2.2 according to sex, with correction for gestational age [38,39]. Nutritional classifications of underweight, stunting and wasting were defined as Z-scores below −2 standard deviations (SD) for WAZ, LAZ, and WLZ, respectively, and for overweight WLZ above +2 SD of the median values of the reference data [40].

Infant feeding information, including breastfeeding and complementary feeding practices, was collected using maternal recall following the WHO global feeding practices indicators [38]. Collected information included duration and type of feeding, age of introduction of complementary feedings, and type of food [38]. The history of the usual food consumption was also collected using the unquantified food frequency questionnaire (FFQ) previously used in similar settings [18,19,41,42], where mothers were asked about the usual infant food consumption during the past seven days.

### 2.3. Data Processing and Statistical Analysis

The Research Electronic Data Capture v8.3.5 was used to capture the data [43]. Z-scores were computed using the Intergrowth-21st and the WHO Anthro [38,39], and values <−3 and >+3 were excluded from analysis due to implausibility from clinical settings. Descriptive statistics were used to present the socio-demographic information, anthropometric measurements, feeding practices and infant HIV exposure. All continuous data were presented as means and standard deviations, with the categorical data represented as frequencies and percentages. The normality of the data was assessed using the Shapiro–Wilk test. Comparisons between HEU and HUU groups were performed using the independent *t*-test (or its non-parametric equivalent Mann–Whitney U test) for continuous variables or the Pearson Chi-squared test for categorical variables. All statistical analyses were performed using R version 4.1.2 program [44] and performed at a 5% level of significance.

### 2.4. Ethical Consideration

The Faculty of Health Sciences Research Ethics Committee (Ref. no.: 294/2017) at the University of Pretoria approved the Siyakhula study. All relevant information was shared with the mothers prior to data collection. Mothers gave consent for themselves and their infants for each study visit, and the Declaration of Helsinki guidelines were followed. This sub-study was approved by the Faculty of Natural and Agricultural Sciences and the Faculty of Health Sciences Research Ethics Committee and the (Ref. no.: NAS063/2020) at the same university.

## 3. Results

### 3.1. Description of the Study Population

A total of 181 mother-infant dyads (86 HEU; 95 HUU) were included in this study. The maternal socio-demographic characteristics are presented in Table 1. Mothers living with and without HIV were similar in terms of employment status, social grants, and access to electricity, while significant differences were found in terms of the mean maternal age (36.9 years ± 8.6 vs. 31.3 ± 6.3 years; *p* < 0.001) and education level (*p* < 0.001). There were no significant differences in the maternal socio-demographic characteristics when comparing the mothers in the overall Siyakhula study and this sub-study).

### 3.2. Birth Characteristics of the Infants

The birth characteristics of the HEU vs. HUU infants are presented in Table 2. More HEU vs. HUU infants were males, but the difference was not statistically significant. Significant differences were found in the mean birth weight (2.84 ± 0.49 kg vs. 3.06 ± 0.51 kg; *p* = 0.005); WAZ (−0.7 ± 0.9 vs. −0.2 ± 1.0; *p* = 0.003); head circumference (33.8 ± 1.8 cm vs. 34.5 ± 1.6 cm; *p* = 0.013) and HCZ (0.3 ± 1.3 vs. 0.7 ± 1.2; *p* = 0.038) of HEU vs. HUU infants. No significant differences were found between HEU vs. HUU infants in terms of the mean length (49.1 ± 4.1 cm vs. 49.9 ± 3.4 cm; *p* = 0.184) and LAZ (0.6 ± 1.4 vs. 0.7 ± 1.5; *p* = 0.804) at birth. In addition, no significant differences were found in the birth characteristics of the HEU vs. HUU infants when comparing the mothers in the overall Siyakhula study and this sub-study.

### 3.3. Feeding Practices

Breastfeeding and complementary feeding of HEU vs. HUU infants are further presented in Table 3. No significant differences were found in the early initiation of breastfeeding between HEU and HUU infants who were breastfed within one hour or after one hour after delivery (*p* = 0.297), based on maternal recall at the time of the study visit. The mean age of breastfeeding cessation was similar in both HEU vs. HUU infants (18.9 ± 15.8 vs. 18.1 ± 15.8 weeks; *p* = 0.778) before 6 months, and the mean age of formula milk introduction (18.4 ± 15.9 vs. 17.4 ± 15.4 weeks; *p* = 0.770). Early introduction of complementary foods before 6 months was common in both groups (16.2 ± 11.0 vs. 12.8 ± 9.3; *p* = 0.118).

**Figure 1:**
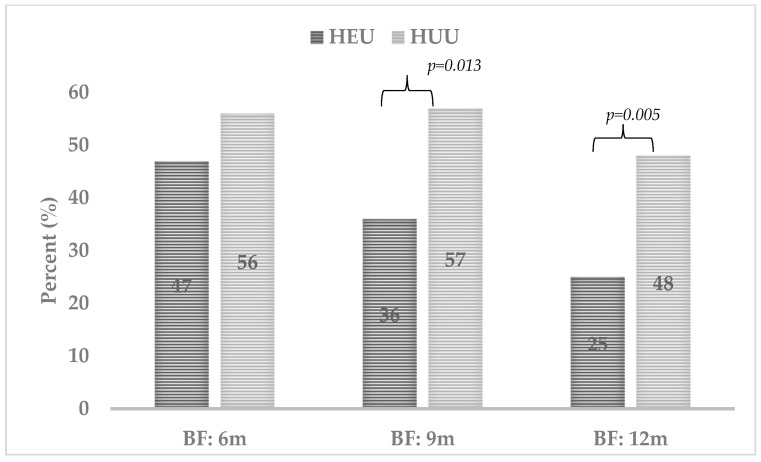
Infant breastfeeding practices by HIV exposure status in the first 12 months of life. Values in *italic* font indicate significant *p*-values (*p* < 0.05). Abbreviations: HEU: HIV-exposed-uninfected; HUU: HIV-unexposed-uninfected. The mixed feeding category was separately calculated, and no comparison tests were performed due to one group having <5 count, which led to volatile results. Pearson’s Chi-square test was used for categorical data to determine the differences in HEU and HUU infants.

The breastfeeding practices amongst HEU and HUU infants at 6, 9, and 12 months are shown in Figure 1. Similar percentages of EBF at birth and breastfeeding at 6 months were observed in the HEU and HUU infants, but significant differences were found at 9 (35.6% vs. 57.3%; *p* = 0.013) and 12 months (24.7% vs. 48.0%; *p* = 0.005).

The foods frequently consumed by infants at 6 and 12 months are presented in Table 4. At 6 months, maizemeal soft porridge (11.4% vs. 10.1%), infant cereal (52.9% vs. 56.2%), and baby food in a jar (34.3% vs. 29.2%) were consumed at least four days per week by HEU vs. HUU infants. At 12 months, carbonated/fizzy drinks (9.3% vs. 14.3%) and sweets/chocolates (22.7% vs. 11.7%) were consumed at least four days per week by HEU vs. HUU infants.

### 3.4. Growth of HEU vs. HUU Infants

The anthropometric measurements, Z-score indices, and nutritional classification of the infants at 6, 9, and 12 months of age by HIV exposure status are presented in Table 5. HEU infants had a significantly lower mean weight compared to HUU infants at 6 months (7.3 ± 0.9 kg vs. 7.8 ± 1.0 kg; *p* = 0.001), and at 9 months (8.3 ± 1.0 vs. 8.8 ± 1.1 kg; *p* = 0.002), also at 12 months the mean weight was lower although this did not reach statistical significance (9.1 ± 1.2 kg vs. 9.4 ± 1.3 kg; *p* = 0.106). The mean WAZ was significantly lower in HEU as compared to HUU infants at 6 (−0.6 ± 1.1 vs. 0.1 ± 1.2; *p* < 0.001), 9 (−0.4 ± 1.1 vs. 0.1 ± 1.1; *p* = 0.003) and 12 months (−0.3 ± 1.1 vs. 0.1 ± 1.2; *p* = 0.022). No significant difference was found in the underweight classification of infants at 6, 9, and 12 months.

The mean length was significantly lower in HEU vs. HUU infants at 6 months (65.3 ± 3.5 cm vs. 66.6 ± 2.8 cm; *p* = 0.014) and 9 months (70.1 ± 3.1 cm vs. 71.2 ± 2.8 cm; *p* = 0.012). The mean LAZ was also significantly lower in HEU vs. HUU infants at 6 months (−0.8 ± 1.4 vs. −0.1 ± 1.2; *p* < 0.001) and 9 months (−0.5 ± 1.4 vs. 0.0 ± 1.3; *p* = 0.023). Furthermore, HEU infants were at a higher risk of being stunted as compared to HUU infants at age 6 months (15.0% vs. 4.6%), although we could not perform significance tests due to low counts in the HUU group. The mean WLZ was significantly lower in HEU infants as compared to HUU infants at 12 months (−0.2 ± 1.2 vs. 0.2 ± 1.2; *p* = 0.020).

## 4. Discussion

Our study showed significant differences in terms of breastfeeding practices and growth between HEU and HUU infants in the second half of the first year of life, with inappropriate infant feeding practices identified. Appropriate feeding practices especially continued breastfeeding, is important from 6 months when a transition occurs from EBF to continued breastfeeding with complementary feeding, making this a critical time for growth monitoring and promotion. Furthermore, breastmilk is the best source of nutrition for infants [3], with inappropriate feeding practices potentially resulting in malnutrition, leading to well-documented increased morbidity and mortality risk [45].

Early cessation of breastfeeding was found in our study, especially in the HEU infants. Lack of knowledge and mothers’ education level is possible contributory reasons for too early cessation of breastfeeding, as reported in another South African study [46]. Our study found the early introduction of complementary foods before 6 months in both groups, with HUU infants given complementary foods at an earlier age than HEU infants, although this difference was not significant. This was similar to another South African study which found the introduction of solids in HEU infants as early as 6 weeks of age [47], while an Ethiopian study found that 58% of HEU infants were not introduced to complementary feeding at the recommended age of 6 months [48].

Almost half (47%) of HEU infants were breastfed at 6 months, signifying the progress of the ART program in terms of breastfeeding promotion [47]. Breastfeeding, however, decreased over time in our study, with mothers stopping breastfeeding their infants at a mean age of 18 weeks, with reasons including the need to return to work (31.4% vs. 43.3%) and insufficient milk or not growing (27.7% vs. 25.4%) in HEU vs. HUU infants. Poor breastfeeding rates in our study may result from cultural practices, lack of knowledge, and previous provision of formula milk through PMTCT programs [49].

Lower breastfeeding rates were found in HEU vs. HUU infants at 9 months, lower than in Kenya [50] but higher than in the South African study in which no HEU infants were breastfed in the year 2009 [49]. Lack of knowledge and HIV stigma need to be addressed to increase the breastfeeding rates in the ART context [51]. The IYCF policy needs to be used to promote, protect and support breastfeeding, even in the context of HIV [52].

The unquantified food frequency questionnaire is simple and quick to administer and can be used to determine the foods frequently consumed [18,53], which may affect the growth of infants. Our results show that HEU (34.3%) and HUU (29.2%) infants were mostly consuming baby food in a jar/pureed at 6 months of age which also increased at 12 months to 45.3% vs. 48.1%, which is similar to other South African data [17,18]. Baby food purée in a jar has been reported to contain insufficient nutrients, such as iron and zinc, which are important for good growth and development [54]. HEU infants consumed more miscellaneous junk food products, such as chips (50.7% vs. 46.8%) and juice concentrate diluted with water (16.0% vs. 6.5%) at least 4 days per week at 12 months, similar to other South African studies [18,47]. The IYCF policy is, thus, very important in emphasizing the correct and timely introduction of complementary foods, which may improve the growth of HEU infants [9].

Our study found poor growth in HEU infants for the first 12 months of their life; similar findings have been reported in HEU infants who experience slower growth than HUU infants over the first 12 months of life resulting from a high risk of opportunistic infections, such as pneumonia and lower respiratory tract infections [5,29,50,55]. The mechanism of higher morbidity in these infants has been suggested to be associated with multifactorial reasons, such as maternal socioeconomic factors, abnormal immunological factors, and maternal nutrition, which have been identified as possible causes of the increased morbidity in HEU infants [56,57]. Furthermore, the maternal use of ART affects the growth of HEU infants [31].

In our study, a lower mean LAZ was found in HEU infants at 6 months, which is similar to another South African study [58], and a lower mean HCZ was also reported in Zimbabwe at 6 months [59]. A lower mean LAZ was also observed at 9 months in HEU infants as compared to HUU infants, similar to other studies in Uganda [60], Rwanda [61], and Botswana [10]. HEU infants were at a higher risk of being stunted (12.5% vs. 7.1%) at 9 months as compared to HUU infants. A higher risk of stunting (20% vs. 10%; *p* < 0.01) was also found in Kenya at 9 months [50]. Stunting has irreversible consequences, yet it is important to identify it at an early age [9,62].

Our study differs from a study conducted in Botswana which found significant differences in HEU vs. HUU infants in terms of the underweight and stunting status at 6–24 months [10]. However, the study only included 37.2% of HEU infants. We found significantly lower mean WLZ in HEU infants as compared to HUU infants at 12 months. Similar findings were reported in another South African study at the age of 12 months [33]. A higher risk of being overweight in infancy might result in higher rates of obesity later in life, which increases the risk of non-communicable diseases [62]. This study found that maternal HIV exposure affects the feeding practices and growth of infants. Breastfeeding practices decreased with age.

The strength of our study lies in detailed infant feeding practices and anthropometric measurements collected by trained field workers to ensure quality control and validity. Further strengths include the repeated anthropometric measurements in infants at 6, 9, and 12 months, a control group (HUU infants), and a similar sample size in the HEU and HUU groups. Limitations of this study include uneven numbers across the time points due to loss of follow-up (some mothers moved away from the study site due to the SA national lockdown during the COVID-19 pandemic, amongst other reasons) and limited sample size. A further limitation is a potential recall bias as the unquantified FFQ asks for food consumed in the past seven days, and no associations were investigated due to the small number of counts in the different consumption frequencies in both the HEU and HUU groups. In addition, our results may only be partially generalizable, taking into account the sociodemographic and geographical context of this study. In addition, the results need to be interpreted with care as adjustment for confounders, such as maternal age, employment status, and education level, which could not be performed due to the low sample size.

## 5. Conclusions

In this study, we compared the feeding practices and growth of infants aged 6 and 12 months exposed and unexposed to maternal HIV infection. HEU infants had lower rates of breastfeeding at 9 and 12 months, and the breastfeeding rates decreased with age. HEU infants had lower LAZ, WAZ, and MUACZ at 6, 9, and 12 months and higher stunting rates compared to the HUU infants. Current findings will inform nutrition policy interventions aimed at educating mothers and caregivers about appropriate complementary feeding in order to promote optimal growth, even in the context of HIV. For future studies, a bigger sample size will be more beneficial, as well as using structured questionnaires to determine nutrient intake from 6 months of age in HEU children.

## Figures and Tables

**Table 1:** Socio-demographic characteristics of the study mothers according to HIV status.

		Study Population	Mothers Living with HIV	Mothers Not Living with HIV	*p*-Value
		(*n* = 181)	(*n* = 86)	(*n* = 95)	
**Age (years)** mean ± SD ^1^		33.9 ± 7.9	36.9 ± 8.6	31.3 ± 6.3	*<0.001*
**Education** *n* (%) ^2^	Formal education, but without school completion ^3^	86 (48.6)	55 (66.3)	31 (33.0)	*<0.001*
	Completed secondary schooling	58 (32.8)	19 (22.9)	39 (41.5)	
	Tertiary education	33 (18.6)	9 (10.8)	24 (25.5)	
**Employment** *n* (%) ^2^	Yes	84 (47.5)	41 (49.4)	43 (45.7)	0.738
**Child support grant** *n* (%) ^2^	Yes	136 (76.8)	62 (74.7)	74 (78.7)	0.649
**Marital status** *n* (%) ^2^	Single	134 (75.7)	60 (72.3)	74 (78.7)	0.412
	Married	43 (24.3)	23 (27.7)	20 (21.3)	
**Access to water** *n* (%) ^2^	Communal tap	40 (22.6)	21 (25.3)	19 (20.2)	0.534
	Inside yard	88 (49.7)	42 (50.6)	46 (48.9)	
	Inside house	49 (27.7)	20 (24.1)	29 (30.9)	
**Access to electricity** *n* (%) ^2^	Yes	165 (93.2)	76 (91.6)	89 (94.7)	0.601
**Access to toilet** *n* (%) ^2^	None ^4^	2 (1.1)	2 (2.4)	0 (0)	0.816
	Pit latrine	60 (33.9)	29 (34.9)	31 (33.0)	
	Flush toilet	115 (65.0)	52 (62.7)	63 (67.0)	

Values in *italic* font indicate significant *p*-values (*p* < 0.05); ^1^ non-normal distributed data; ^2^ excludes missing numbers; ^3^ formal education = includes any primary and secondary schooling; ^4^ none: not considered in the calculation. Mann–Whitney U test was used for continuous non-normally distributed data; Pearson’s Chi-square test was used for categorical data to determine the differences in mothers living with HIV and mothers not living with HIV.

**Table 2:** Gestational age, sex, and anthropometric measurements and indices of HIV-exposed-uninfected and HIV-unexposed-uninfected infants at birth.

		Study Population	HEU Infants	HUU Infants	*p*-Value
		(*n* = 181)	(*n* = 86)	(*n* = 95)	
**Gestational age (weeks)** ^1,2^		38.2 ± 1.7	38.2 ± 1.5	38.3 ± 1.8	0.293
**Infant sex** *n* (%)	Male	105 (58.0)	54 (62.8)	51 (53.7)	0.276
**Birth body measurements** ^1^	Weight (kg) ^3^	2.95 ± 0.51	2.84 ± 0.49	3.06 ± 0.51	*0.005*
	Length (cm) ^2^	49.5 ± 3.8	49.1 ± 4.1	49.9 ± 3.4	0.184
	Head circumference (cm) ^2^	34.1 ± 1.7	33.8 ± 1.8	34.5 ± 1.6	*0.013*
**Birth Z-scores indices** ^1,3,4^	Weight-for-age	−0.5 ± 1.0	−0.7 ± 0.9	−0.2 ± 1.0	*0.003*
	Length-for-age	0.6 ± 1.5	0.6 ± 1.4	0.7 ± 1.5	0.804
	Head circumference-for-age	0.5 ± 1.3	0.3 ± 1.3	0.7 ± 1.2	*0.038*

Values in *italic* font indicate significant *p*-values (*p* < 0.05). Abbreviations: HEU: HIV-exposed-uninfected (born to mothers living with HIV); HUU: HIV-unexposed-uninfected (born to mothers not living with HIV); ^1^ data presented as mean ± SD; ^2^ non-normal distributed data; ^3^ normal distributed data; ^4^ the birth Z-scores indices sex-normalized were computed using INTERGROWTH-21st software, using gestation-adjusted age for preterm infants. Independent t-test was used for continuous normally distributed data, and the Mann–Whitney U test was used for continuous non-normally distributed data; Pearson’s Chi-square test was used for categorical data to determine the differences in HEU and HUU infants.

**Table 3:** Feeding practices of HEU and HUU infants before 6 months based on maternal recall.

		HEU Infants	HUU Infants	*p*-Value
		*n* = 86	*n* = 95	
**Initiation of breastfeeding** *n* (%) ^1,2^	<1 h after birth	40 (46.5)	55 (57.9)	0.297
	>1 h after birth	39 (45.3)	33 (34.7)	
	Never breastfed	7 (8.2)	7 (7.4)	
**Baby received liquids/foods other than breastmilk/formula milk before age 6 months** *n* (%) ^1,2^	Yes	60 (69.8)	76 (80.9)	0.120
**Breastfeeding cessation age (weeks)** ^1,2,3^		18.9 ± 15.8	18.1 ± 15.8	0.778
**Formula milk introduction mean age (weeks)** ^1,2,3^		18.4 ± 15.9	17.4 ± 15.4	0.770
**Type of formula milk** *n* (%) ^1,2^	Commercial cow’s milk-based formula	55 (93.2)	49 (84.5)	n/a
	Others ^4^	4 (6.8)	9 (15.5)	
**Main reason for introducing formula milk** *n* (%) ^1,2^	Return to work	17 (31.5)	29 (43.3)	0.281
	Insufficient milk/baby not growing	15 (27.8)	17 (25.4)	
	Convenience	6 (11.1)	10 (14.9)	
	Baby/mother unwell	16 (29.6)	11 (16.4)	
**Complementary feeding introduction (weeks)** ^1,2,3^		16.2 ± 11.0	12.8 ± 9.3	0.118
**First liquid introduced** *n* (%) ^1,2^	Water	71 (91.0)	85 (92.4)	0.966
	Others ^5^	7 (9.0)	7 (7.6)	
**First solid food introduced** *n* (%) ^1,2^	Mabelle/maize meal soft porridge	72 (83.7)	72 (75.8)	0.256
	Others ^6^	14 (16.3)	23 (24.2)	

Abbreviations: HEU: HIV-exposed-uninfected; HUU: HIV-unexposed-uninfected; n/a: not applicable (no comparisons were performed due to one of the groups having less than five count leading to volatile results. ^1^ result based on maternal recall and *n* numbers vary as mothers with missing information were excluded; ^2^ data presented as mean ± SD; ^3^ non-normal distributed data; ^4^ others include lactose-free cow’s milk and soy-based formula; ^5^ others include tea and juice; ^6^ others include baby cereal and instant porridge; Mann–Whitney U test used for continuous non-normally distributed data; Pearson’s Chi-square test used for categorical data to determine the differences in HEU and HUU infants. Significant *p*-values were defined as *p* < 0.05.

**Table 4:** Usual intake of food items by 6 and 12 months old HEU compared to HUU infants determined by quantified food frequency questionnaire (%).

Foods	Consumption at Age 6 Months (%) ^1^						Consumption at Age 12 Months (%) ^2^					
	Most Days ^3^		Once a Week		Never		Most Days ^3^		Once a Week		Never	
	HEU Infants	HUU Infants	HEU Infants	HUU Infants	HEU Infants	HUU Infants	HEU Infants	HUU Infants	HEU Infants	HUU Infants	HEU Infants	HUU Infants
**Starches**												
Maizemeal porridge—soft	11.4	10.1	7.1	3.4	81.4	86.5	49.3	42.9	6.7	15.6	44.0	41.6
Infant cereal	52.9	56.2	2.9	6.7	44.3	37.1	38.7	46.8	9.3	6.5	52.0	46.8
Instant porridge	10.0	11.2	2.9	0	87.1	88.8	28.0	16.9	14.7	13.0	57.3	70.1
Bread	4.3	0	1.4	4.5	94.3	95.5	56.0	54.5	28.0	27.3	16.0	18.2
Rice	1.4	0	2.9	1.1	95.7	98.9	17.3	18.2	42.7	37.7	40.0	44.2
Potato	15.7	11.2	15.7	13.5	68.6	75.3	54.7	42.9	36.0	44.2	9.3	13.0
**Dairy products**												
Fresh, fermented or powder milk	0	0	0	2.2	100	97.8	13.3	19.5	22.7	15.6	64.0	64.9
Yoghurt/dairy snack for baby	7.1	1.1	4.3	7.9	88.6	91.0	33.3	31.2	26.7	39.0	40.0	29.9
**Animal foods/meat products**												
Red meat	0	0	2.9	0	97.1	100	13.3	10.4	28.0	32.5	58.7	57.1
Liver	0	0	2.9	3.4	97.1	96.6	10.7	11.7	33.3	37.7	56.0	50.6
Chicken	0	5.6	4.3	3.4	95.7	91.0	36.0	40.3	32.0	27.3	32.0	32.5
Fish	1.4	0	2.9	2.2	95.7	97.8	12.0	11.7	34.7	32.5	53.3	55.8
Eggs	4.3	3.4	2.9	6.7	92.9	89.9	38.7	27.3	33.3	44.2	28.0	28.6
**Vegetables**												
Any vegetables ^4^	9.0	8.7	14.5	10.1	76.8	80.9	48.0	51.9	40.0	31.2	12	16.9
Orange ^5^	87.5	88.2	n/a	n/a	n/a	n/a	70.7	68.8	n/a	n/a	n/a	n/a
Dark-green leafy ^6^	6.3	11.8	n/a	n/a	n/a	n/a	17.3	12.9	n/a	n/a	n/a	n/a
Red/yellow ^7^	6.2	0	n/a	n/a	n/a	n/a	0	0	n/a	n/a	n/a	n/a
**Fruits**												
Any fruits ^8^	11.4	6.8	11.4	9.1	77.1	84.1	64.0	59.17	24.0	31.2	12.0	9.1
Orange ^9^	28.6	18.8	n/a	n/a	n/a	n/a	58.7	63.8	n/a	n/a	n/a	n/a
Green ^10^	28.6	12.5	n/a	n/a	n/a	n/a	29.3	36.2	n/a	n/a	n/a	n/a
Red/yellow ^11^	42.8	68.7	n/a	n/a	n/a	n/a	0	0	n/a	n/a	n/a	n/a
**Food items added to porridge**												
Salt	5.7	4.5	2.9	3.4	91.4	92.1	62.7	54.5	9.3	14.3	28.0	31.2
Oil	7.1	5.6	5.7	5.6	87.1	88.8	50.7	37.7	20.0	22.1	29.3	40.3
Margarine	8.6	7.9	10.0	6.7	81.4	85.4	42.7	29.9	26.7	28.6	30.7	41.6
Peanut butter	4.3	3.4	2.9	6.7	92.9	89.9	29.3	35.1	20.0	16.9	50.7	48.1
**Miscellaneous**												
Sweets/chocolates	1.4	0	7.1	5.6	91.4	94.4	22.7	11.7	29.3	29.9	48.0	58.4
Kids tea	4.3	2.2	4.3	3.4	91.4	94.4	46.7	35.1	18.7	15.6	34.7	49.4
Black/English tea	0	0	0	0	100	100	10.7	6.5	4.0	7.8	85.3	85.7
Chips	1.4	2.2	12.9	4.5	85.7	93.3	50.7	46.8	34.7	32.5	14.7	20.8
Carbonated/fizzy drinks	1.4	0	2.9	4.5	95.7	95.5	9.3	14.3	32.0	18.2	58.7	67.5
Fruit juice	4.3	5.7	7.2	8.0	88.5	86.3	21.3	36.4	34.7	28.6	44.0	35.1
Baby food in a jar/pureed	34.3	29.2	11.4	14.6	54.3	56.2	45.3	48.1	33.3	22.1	21.3	29.9
Juice concentrate	1.4	1.1	2.9	3.4	95.7	95.5	16.0	6.5	18.7	24.7	65.3	68.8

Abbreviations: HEU: HIV-exposed-uninfected; HUU: HIV-unexposed-uninfected; no comparisons were performed due to one of the groups having less than five counts leading to volatile results. ^1^ 6-months: HEU = 70, HUU = 89; ^2^ 12 months: HEU = 75, HUU = 77; ^3^ %: the categories ‘every day’ and ‘most days’ are grouped together; n/a: not applicable; ^4^ excludes infants who did not consume any vegetables; ^5^ orange colored vegetables (carrots, butternut, pumpkin, sweet potato); ^6^ dark-green-leafy colored vegetables (spinach, butternut leaves, cabbage); ^7^ red/yellow colored vegetables (tomatoes, beetroot, corn); ^8^ excludes infants who did not consume any fruits; ^9^ orange colored fruits (mangoes, peaches, oranges, mandarins); ^10^ green colored fruits (apples, grapes, avocado, pear) and ^11^ red/yellow colored fruits (banana, pineapple, watermelon, strawberry).

**Table 5:** Anthropometric measurements, Z-score indices, and nutritional classifications of the infants between 6–12 months of life by HIV exposure status.

	Age 6 Months			Age 9 Months			Age 12 Months		
	HEU Infants	HUU Infants	*p*-Value	HEU Infants	HUU Infants	*p*-Value	HEU Infants	HUU Infants	*p*-Value
	(*n* = 86)	(*n* = 95)		(*n* = 80)	(*n* = 86)		(*n* = 75)	(*n* = 80)	
**Anthropometric measurements**									
**Weight (kg)** ^1^	7.3 ± 0.9 ^2^	7.8 ± 1.0 ^2^	*0.001*	8.3 ± 1.0 ^3^	8.8 ± 1.1 ^3^	*0.002*	9.1 ± 1.2 ^2^	9.4 ± 1.3 ^2^	0.106
**Length (cm)** ^1^	65.3 ± 3.5 ^2^	66.6 ± 2.8 ^2^	*0.014*	70.1 ± 3.1 ^3^	71.2 ± 2.8 ^3^	*0.012*	74.4 ± 3.1 ^3^	74.5 ± 2.7 ^3^	0.704
**Head circumference (cm)** ^1^	43.5 ± 1.6 ^2^	43.9 ± 1.6 ^2^	0.106	45.3 ± 1.4 ^3^	45.4 ± 1.7 ^3^	0.621	46.5 ± 1.6 ^3^	46.6 ± 1.6 ^3^	0.655
**Mid-upper-arm-circumference (cm)** ^1^	14.6 ± 1.3 ^2^	15.2 ± 1.1 ^2^	*0.002*	15.2 ± 1.2 ^2^	15.7 ± 1.5 ^2^	*0.026*	15.6 ± 1.2 ^2^	16.0 ± 1.3 ^2^	0.075
**Z-score indices**									
**Weight-for-age Z-score** ^1,4^	−0.6 ± 1.1 ^3^	0.1 ± 1.2 ^3^	*<0.001*	−0.4 ± 1.1 ^3^	0.1 ± 1.1 ^3^	*0.003*	−0.3 ± 1.1 ^2^	0.1 ± 1.2 ^2^	*0.022*
**Length-for-age Z-score** ^1,4^	−0.8 ± 1.4 ^3^	−0.1 ± 1.2 ^3^	*<0.001*	−0.5 ± 1.4 ^3^	0.0 ± 1.3 ^3^	*0.023*	−0.4 ± 1.3 ^3^	−0.2 ± 1.1 ^3^	0.308
**Weight-for-length Z-score** ^1,4^	−0.1 ± 1.2 ^3^	0.2 ± 1.1 ^3^	0.074	−0.1 ± 1.2 ^3^	0.2 ± 1.1 ^3^	0.098	−0.2 ± 1.2 ^3^	0.2 ± 1.2 ^3^	*0.020*
**Head circumference-for-age Z-score** ^1,4^	0.5 ± 1.2 ^3^	0.9 ± 1.2 ^3^	*0.019*	0.6 ± 1.2 ^3^	0.8 ± 1.0 ^3^	0.331	0.6 ± 1.2 ^3^	0.9 ± 1.1 ^3^	0.069
**Mid-upper-arm-circumference-for-age Z-score** ^1,4^	0.5 ± 1.1 ^3^	1.0 ± 0.9 ^3^	*<0.001*	0.7 ± 1.0 ^3^	1.1 ± 1.1 ^3^	*0.013*	0.8 ± 1.1 ^3^	1.3 ± 1.1 ^3^	*0.025*
**Nutritional classifications**									
**Underweight** *n* (%) ^5^	7 (8.9)	3 (3.4)	n/a	7 (8.8)	2 (2.4)	n/a	4 (5.5)	4 (5.1)	n/a
**Stunted** *n* (%) ^6^	12 (15.0)	4 (4.6)	n/a	10 (12.5)	6 (7.1)	0.297	9 (12.3)	3 (3.8)	n/a
**Wasted** *n* (%) ^7^	3 (3.7)	2 (2.3)	n/a	5 (6.2)	3 (3.6)	n/a	4 (5.5)	3 (3.9)	n/a
**Overweight** *n* (%) ^8^	4 (4.9)	6 (6.8)	n/a	4 (5.0)	3 (3.6)	n/a	4 (5.5)	6 (7.8)	n/a
**Acute malnutrition** *n* (%) ^9^	1 (1.3)	0 (0)	n/a	1 (1.2)	0 (0)	n/a	1 (1.4)	0 (0)	n/a
**Macrocephalus** *n* (%) ^10^	8 (10.0)	17 (19.3)	0.140	10 (12.5)	10 (12.2)	>0.999	10 (13.7)	13 (16.9)	0.753

Values in *italics* font indicate significant *p*-values. Abbreviations: HEU: HIV-exposed-uninfected; HUU: HIV-unexposed-uninfected; n/a: not applicable (no comparisons were performed due to one of the groups having less than five counts leading to volatile results. ^1^ data presented as mean ± SD; ^2^ non-normal distributed data; ^3^ normal distributed data; ^4^ sex-normalized Z-scores indices at age 6–12 months were computed using World Health Organization Anthro software of 2010, using gestation-adjusted age for preterm infants; ^5^ underweight from weight-for-age Z-scores < −2; ^6^ stunted from length-for-age Z-scores < −2; ^7^ wasted from weight-for-length Z-scores (WLZ) < −2; ^8^ overweight from WLZ > +2; ^9^ acute-malnutrition from mid-upper-arm-circumference Z-scores < −2; ^10^ macrocephalus from head circumference-for-age Z-scores > +2. Independent t-test was used for continuous normally distributed data, and the Mann–Whitney U test was used for continuous non-normally distributed data; Pearson’s Chi-square test was used for categorical determine the differences in HEU and HUU infants (*p* < 0.05).

## Data Availability

Data are available on request from the corresponding author, due to the University of Pretoria policy on data publication.
